# Comprehensive predictive model for cerebral microbleeds: integrating clinical and biochemical markers

**DOI:** 10.3389/fnins.2024.1429088

**Published:** 2024-12-13

**Authors:** Lijing Wang, Yao Li, Yadong Hu, Li Ling, Nan Jia, Yajing Chen, Yanan Meng, Ye Jiang, Ning Li

**Affiliations:** ^1^Department of Neurology, Affiliated Hospital of Hebei University, Baoding, China; ^2^Department of Neurosurgery, Affiliated Hospital of Hebei University, Baoding, China

**Keywords:** cerebral microbleeds (CMBs), predictive modeling, risk assessment, cerebral small vessel disease (CSVD), statistical analysis, neuroimaging, biomarkers

## Abstract

**Background:**

Cerebral Microbleeds (CMBs) serve as critical indicators of cerebral small vessel disease and are strongly associated with severe neurological disorders, including cognitive impairments, stroke, and dementia. Despite the importance of diagnosing and preventing CMBs, there is a significant lack of effective predictive tools in clinical settings, hindering comprehensive assessment and timely intervention.

**Objective:**

This study aims to develop a robust predictive model for CMBs by integrating a broad range of clinical and laboratory parameters, enhancing early diagnosis and risk stratification.

**Methods:**

We analyzed extensive data from 587 neurology inpatients using advanced statistical techniques, including Least Absolute Shrinkage and Selection Operator (LASSO) and logistic regression. Key predictive factors such as Albumin/Globulin ratio, gender, hypertension, homocysteine levels, Neutrophil to HDL Ratio (NHR), and history of stroke were evaluated. Model validation was performed through Receiver Operating Characteristic (ROC) curves and Decision Curve Analysis (DCA).

**Results:**

The model demonstrated strong predictive performance with significant clinical applicability. Key predictors identified include the Albumin/Globulin ratio, homocysteine levels, and NHR, among others. Validation metrics such as the area under the ROC curve (AUC) and decision curve analysis confirmed the model’s utility in predicting CMBs, highlighting its potential for clinical implementation.

**Conclusion:**

The comprehensive predictive model developed in this study offers a significant advancement in the personalized management of patients at risk for CMBs. By addressing the gap in effective predictive tools, this model facilitates early diagnosis and targeted intervention, potentially reducing the incidence of stroke and cognitive impairments associated with cerebral microbleeds. Our findings advocate for a more nuanced approach to cerebrovascular disease management, emphasizing the importance of multi-factorial risk profiling.

## Introduction

Cerebral Microbleeds (CMBs), as prominent indicators of small vessel disease, are closely associated with a spectrum of severe neurological disorders, including cognitive impairments, stroke, and dementia (Lau et al., 2017; Gorelick and Farooq, 2016; Martinez-Ramirez et al., 2014), particularly in the context of cerebral amyloid angiopathy (CAA) and Alzheimer’s disease (Janelidze et al., 2016; Charidimou et al., 2017; Greenberg and Charidimou, 2018). The presence of CMBs not only significantly elevates the risk of both hemorrhagic and ischemic strokes (Wilson et al., 2019; Smith et al., 2017) but also plays a crucial role in unveiling and understanding the pathophysiology of cerebrovascular diseases (Wardlaw et al., 2013; Shi and Wardlaw, 2016; Lu et al., 2021). CMBs often remain asymptomatic in their initial stages. The diagnostic capability of Magnetic Resonance Imaging (MRI) in early lesion detection is limited, posing challenges in early identification and intervention of CMBs in clinical practice.

Accurate prediction of CMBs is essential for early intervention and improving the management of Cerebral Small Vessel Disease (CSVD) and its complications. However, an effective predictive model for CMBs is notably lacking in clinical settings, limiting comprehensive assessment and timely intervention of this critical neuro-radiological marker.

This study presents a novel predictive model for CMBs by integrating multidimensional clinical and biochemical markers into the prediction framework. Compared to conventional models that primarily rely on imaging features and clinical variables, this approach offers a more comprehensive assessment. Traditional predictive models typically emphasize standard risk factors, such as age and hypertension (Cannistraro et al., 2019; Gottesman and Seshadri, 2022), but often lack systematic incorporation of biochemical markers. In this model, biomarkers such as sex, hypertension, homocysteine levels, neutrophil-to-HDL ratio (NHR), history of stroke, and albumin-to-globulin (A/G) ratio were incorporated, all of which have been shown to correlate significantly with the occurrence of CMBs (Cao et al., 2021; Dobrynina et al., 2018; Casimir and Duchateau, 2011; Koep et al., 2023; Guo et al., 2023; Wang et al., 2023; Shoamanesh et al., 2015). Specifically, NHR is widely used to assess inflammatory status and has been linked to increased risk of cerebrovascular events (Yu et al., 2023; Wang et al., 2022). The A/G ratio, reflecting inflammation and nutritional status, is associated with ischemic event outcomes (Wang et al., 2023; Yao et al., 2023) and may offer insights into cerebrovascular health. Additionally, elevated homocysteine levels and history of stroke have been consistently identified as major risk factors for CSVD in multiple studies (Cao et al., 2021; Nam et al., 2019; Ji et al., 2020). Other factors, such as sex and hypertension, are critical for CMB prediction due to their roles in vascular function regulation (Shoamanesh et al., 2015). Including these factors in the model enhances the ability to detect CMBs early and offers a new tool for personalized risk assessment, addressing the limitations of existing prediction tools that lack biochemical marker integration.

This study aims to develop a comprehensive predictive model for CMBs by analyzing extensive clinical and laboratory data from 587 inpatients in the neurology department. Incorporating a wide array of factors including cranial MRI findings, age, gender, history of hypertension, diabetes, along with blood biochemical indicators, we endeavor to construct an efficient tool for assessing the risk of CMBs.

We employed advanced statistical methods, including Least Absolute Shrinkage and Selection Operator (LASSO) and logistic regression techniques, to deeply analyze these data, aiming to identify risk factors closely associated with the development of CMBs. Our study not only reveals a series of significant predictive factors correlated with CMBs but also demonstrates their substantial value in clinical application. Furthermore, our model efficiently predicts the presence of CMBs, providing clinicians with a powerful tool for early diagnosis and intervention. These findings offer new perspectives for further research and the formulation of future therapeutic strategies.

The outcomes of this research signify a significant advancement in the field of personalized medicine and targeted intervention, particularly in neurology. Our model holds promise in improving the identification and management of patients at high risk for CMBs, potentially reducing the incidence of stroke and cognitive impairments associated with CMBs.

## Methods

### Study population and design

In this study, a retrospective approach was adopted, primarily informed by the necessity of analyzing pre-existing patient records. The research was conducted at the Neurology Department of the Affiliated Hospital of Hebei University, spanning from January 2020 to June 2022. We meticulously compiled data from existing medical records, a methodological choice that facilitated an in-depth analysis of pre-collected data. The large sample size inherent to this retrospective analysis augmented the statistical robustness of our findings. To ensure a representative and relevant sample, we established specific inclusion and exclusion criteria based on MRI sequence quality and clinical conditions. Inclusion criteria were: (1) patients aged 55 years or older; and (2) availability of comprehensive cranial MRI sequences, including T1-weighted axial, T2-weighted axial, T2-weighted fluid-attenuated inversion recovery (FLAIR), and axial susceptibility-weighted images. Exclusion criteria were: (1) cases with suboptimal MRI image quality; (2) patients with significant stroke events potentially affecting accurate CMB assessment; (3) patients with severe comorbidities, such as acute myocardial infarction, advanced heart failure, critical infections, severe respiratory insufficiency, end-stage renal or hepatic disease, or active tumors, which could skew laboratory results; (4) patients with conditions of non-vascular origins of CSVD, including multiple sclerosis, intracranial tumors, or demyelinating diseases of the central nervous system; and (5) instances of insufficient clinical or laboratory data. This retrospective study was conducted with rigorous adherence to ethical research practices. Emphasizing the meticulous collection and analysis of pre-existing patient data and records, we ensured the privacy and confidentiality of all participants. All patient records were anonymized prior to analysis, with personal identifiers removed to uphold strict confidentiality. Furthermore, considering the study’s retrospective nature and the use of de-identified data, the Institutional Review Board (IRB) of the Affiliated Hospital of Hebei University granted an exemption from informed consent. This decision was based on the absence of direct patient interaction and alignment with ethical and legal standards, ensuring compliance with national and international data protection regulations, including the principles of the Declaration of Helsinki. The IRB thoroughly reviewed and approved our data protection measures (Approval Number HDFYLL-KY-2023-060), affirming that the study met the highest standards of patient data security and ethical research practices. This commitment to data security and ethical compliance highlights our dedication to maintaining participant privacy while contributing to cerebral small vessel disease research.

### MRI acquisition and assessment for CMBs

In this study, participants underwent brain MRI examinations using a 1.5 T MRI scanner (Siemens, Munich, Germany) to assess the presence of CMBs. The standardized MRI protocol was tailored to optimize the detection of CMBs and included axial T1-weighted, T2-weighted fluid-attenuated inversion recovery (FLAIR), and most importantly, axial susceptibility-weighted imaging (SWI) sequences. Imaging parameters were meticulously set to maximize the visibility of CMBs: a slice thickness of 5 mm with a 1-mm interslice gap was used across sequences. The SWI sequence, critical for identifying CMBs, had repetition time (TR)/echo time (TE) parameters set at 49/40 ms.

Participants were categorized into CMBs and non-CMBs groups based on SWI sequence results, ensuring both groups were assessed under identical imaging criteria. CMBs are identified as small, rounded, well-defined hypointense lesions on SWI sequences, contrasting with the surrounding brain parenchyma (Wardlaw et al., 2013; Duering et al., 2023). These lesions, typically less than 10 mm in diameter, are indicative of hemosiderin deposits resulting from microhemorrhages. To minimize misclassification, CMBs were distinguished from other hypointense lesions (e.g., iron deposits, calcifications, or vascular structures) based on location and morphological features. Lesions in areas prone to artifact, such as near the skull base or sinuses, were carefully reviewed to ensure accurate identification. The identification and precise localization of CMBs were independently conducted by two experienced neuroimaging specialists, Yan Hou and Huan Zhou, who were blinded to the clinical data of the participants. This was to minimize assessment bias. The interrater reliability of their assessments was quantified using the intraclass correlation coefficient (ICC). The achieved ICC value of 0.85 indicated a substantial agreement, reflecting a high level of consistency between the raters in identifying CMBs. The ICC of 0.85 demonstrates robust interrater reliability, underscoring the reproducibility of our CMB identification approach. This reliability is critical to ensure that group classification is consistent and valid for subsequent analysis. This comprehensive approach to MRI acquisition and assessment ensures an accurate identification of CMBs, thereby contributing significantly to the understanding of cerebral small vessel disease in the studied population.

### Clinical blood biochemistry analysis

Our retrospective evaluation involved compiling an extensive array of clinical blood biochemistry markers from our patient cohort. This comprehensive dataset included a wide range of indicators such as full blood counts, markers of renal function, electrolytes, coagulation profiles, both random and fasting glucose levels, liver enzymes, lipid panels, cardiac markers, thyroid function tests, and homocysteine levels. In total, 81 diverse laboratory parameters were analyzed, providing a detailed insight into each patient’s health status and offering a holistic view of their clinical profiles.

### Comprehensive clinical evaluation

Each participant in this study underwent a thorough clinical evaluation, encompassing the collection of demographic data like age and gender, coupled with an exhaustive medical history review. The assessment particularly focused on key health indicators, notably hypertension, diabetes, hypercholesterolemia, along with histories of carotid artery disease and stroke. The definitions for these conditions were stringently applied as follows: Hypertension was classified as a systolic blood pressure ≥ 140 mmHg, diastolic pressure ≥ 90 mmHg, or current antihypertensive medication use. Diabetes was identified by fasting glucose levels ≥7.0 mmol/L, 2-h postprandial glucose >11.1 mmol/L, or active treatment with antidiabetic drugs. Hypercholesterolemia was determined by total or LDL cholesterol levels exceeding normal limits. Carotid atherosclerosis was defined based on a history of the disease or evidence of plaque or increased intima-media thickness in carotid ultrasound imaging.

### Statistical methodology

Our analysis encompassed a patient cohort of 587 individuals, randomly divided into a training set (412 patients) and a validation set (175 patients), following a 7:3 allocation ratio. This division facilitated a robust model development and subsequent validation process. In line with established practices for predictive modeling in medical research, continuous variables were transformed into categorical ones. This standard conversion enhances interpretability and bolsters the model’s applicability across various clinical settings (Bennette and Vickers, 2012; Barrio et al., 2017).

Missing data were handled based on the extent of missingness in each variable. Variables with a high proportion of missing values were excluded from analysis. For variables with low proportions of missing data, multiple imputation was used to fill in missing values, maintaining dataset integrity and minimizing potential bias.

For initial data analysis, categorical variables were summarized as frequencies and percentages, and baseline characteristics between groups were compared using *χ*^2^ or Fisher’s exact tests, as appropriate. Variable selection within the training dataset was conducted via the LASSO regression, using the “glmnet” package in R software (version 4.3.0). Variables entered into the LASSO model were pre-selected based on clinical relevance and prior literature linking them to cerebrovascular health and CMB risk. This approach ensured that only markers with demonstrated associations were included, making the selection process both data-driven and evidence-based. The optimal regularization parameter (lambda) was determined using the lambda.1se criterion, balancing model simplicity and predictive accuracy.

Subsequently, the variables identified by the LASSO model as significant were further analyzed using binary logistic regression to determine independent predictors, which were then incorporated into a nomogram. The nomogram visually represents CMB risk by assigning weighted scores to each predictor based on its logistic regression coefficient, allowing clinicians to estimate a patient’s risk of developing CMBs.

The efficacy of the nomogram was assessed through Receiver Operating Characteristic (ROC) Curve analysis, focusing on the Area Under the Curve (AUC) as a measure of the model’s predictive capacity. Calibration plots were generated to compare predicted probabilities with actual outcomes, evaluating the model’s precision, while Decision Curve Analysis (DCA) was conducted to assess the clinical utility of the model by examining net benefits across different threshold probabilities.

All statistical analyses were conducted using R software (version 4.3.0), with a predefined significance threshold of *p* < 0.05 (two-tailed) for inferential statistics, aligning with standard practices in medical research.

## Results

### Baseline characteristics

**Figure 1:**
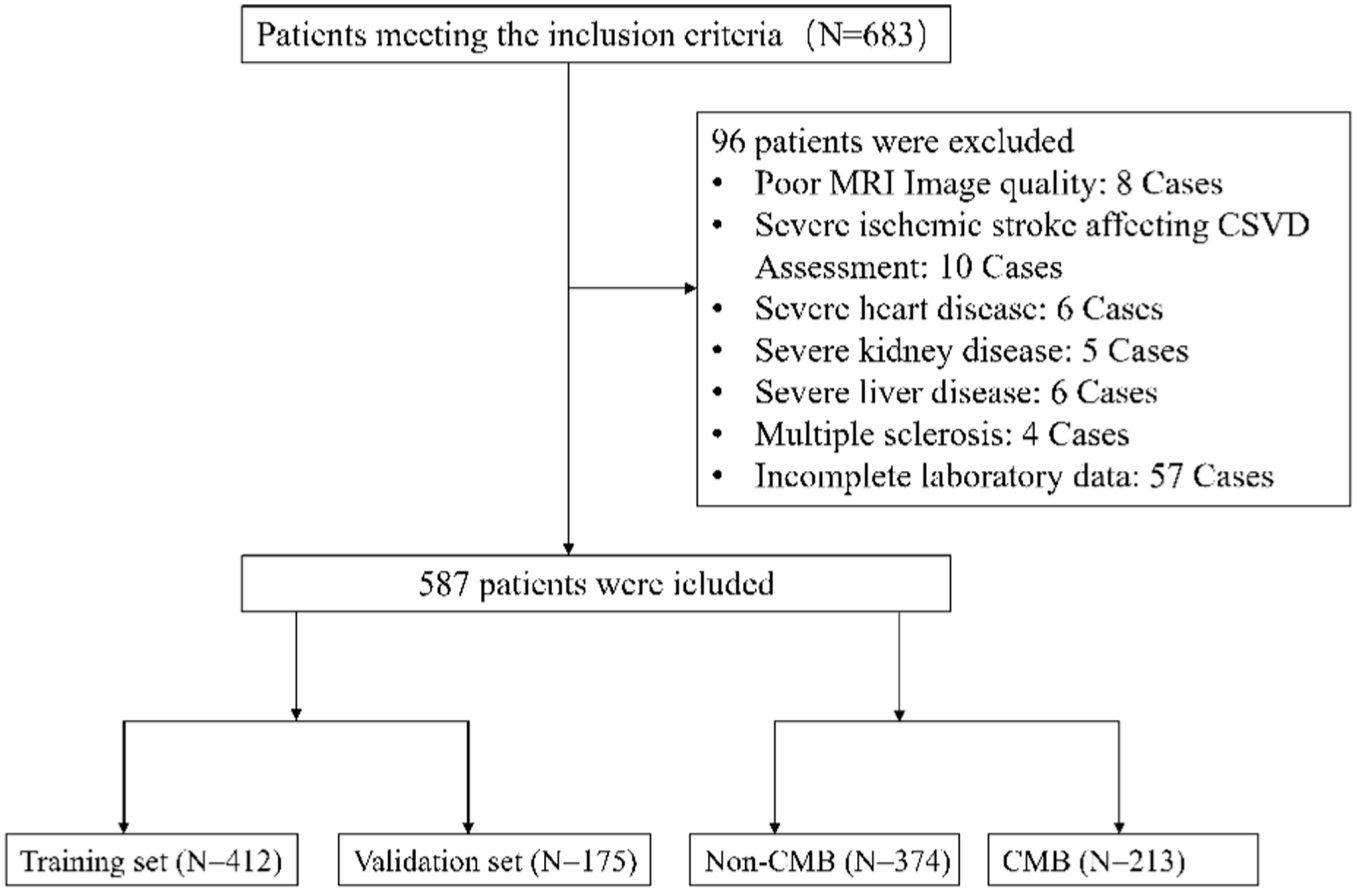
This flowchart depicts the patient selection process for the study.

From the onset of our study in January 2020 until its conclusion in June 2022, an initial group of 683 participants, meeting the set inclusion criteria, was assembled. Subsequent meticulous evaluation led to the exclusion of 96 individuals who met our exclusion criteria. Consequently, this refined the study population to 587 eligible patients for comprehensive data analysis, as graphically represented in Figure 1. Within this cohort, 412 participants were allocated to the training set and 175 to the validation set, as detailed in Table 1, which outlines their baseline characteristics, segregating them into CMBs and non-CMBs groups.

**Table 1:** Baseline characteristics: comparing CMBs patients with non-CMBs patients.

Variables	Total (*n* = 587)	Non-CMBs (*n* = 374)	CMBs (*n* = 213)	*p*
Age, n (%)				0.003
≤63	303 (52)	211 (56)	92 (43)	
>63	284 (48)	163 (44)	121 (57)	
Hypertension, n (%)				<0.001
Normal	189 (32)	161 (43)	28 (13)	
Level 1	109 (19)	73 (20)	36 (17)	
Level 2	38 (6)	22 (6)	16 (8)	
Level 3	251 (43)	118 (32)	133 (62)	
Gender, n (%)				<0.001
Female	307 (52)	229 (61)	78 (37)	
Male	280 (48)	145 (39)	135 (63)	
Stroke, n (%)				<0.001
No	443 (75)	319 (85)	124 (58)	
Yes	144 (25)	55 (15)	89 (42)	
Carotid Atherosclerosis, n (%)				<0.001
No	245 (42)	181 (48)	64 (30)	
Yes	342 (58)	193 (52)	149 (70)	
Creatinine (μmol/L), n (%)				<0.001
≤77	474 (81)	330 (88)	144 (68)	
>77	113 (19)	44 (12)	69 (32)	
Homocysteine, n (%)				<0.001
≤20	449 (76)	311 (83)	138 (65)	
>20	138 (24)	63 (17)	75 (35)	
Neutrophil to HDL Ratio, n (%)				<0.001
≤4.60	380 (65)	269 (72)	111 (52)	
>4.60	207 (35)	105 (28)	102 (48)	
White Blood Cell Count (×109/L), n (%)				0.003
≤6.73	294 (50)	205 (55)	89 (42)	
>6.73	293 (50)	169 (45)	124 (58)	
Neutrophil Count (×109/L), n (%)				0.02
≤4.36	295 (50)	202 (54)	93 (44)	
>4.36	292 (50)	172 (46)	120 (56)	
Monocyte Count (×109/L), n (%)				0.02
≤0.44	295 (50)	202 (54)	93 (44)	
>0.44	292 (50)	172 (46)	120 (56)	
Uric Acid (μmol/L), n (%)				0.019
≤298	298 (51)	204 (55)	94 (44)	
>298	289 (49)	170 (45)	119 (56)	
Calcium (mmol/L), n (%)				0.002
≤2.32	310 (53)	179 (48)	131 (62)	
>2.32	277 (47)	195 (52)	82 (38)	
International Normalized Ratio, n (%)				0.012
≤0.98	306 (52)	210 (56)	96 (45)	
>0.98	281 (48)	164 (44)	117 (55)	
Fibrinogen (g/L), n (%)				0.027
≤2.88	296 (50)	202 (54)	94 (44)	
>2.88	291 (50)	172 (46)	119 (56)	
Apolipoprotein A1 (g/L), n (%)				0.003
≤1.03	296 (50)	171 (46)	125 (59)	
>1.03	291 (50)	203 (54)	88 (41)	
Apolipoprotein B100 (g/L), n (%)				0.001
≤0.79	297 (51)	170 (45)	127 (60)	
>0.79	290 (49)	204 (55)	86 (40)	
Apolipoprotein E (mg/L), n (%)				0.002
≤37	296 (50)	170 (45)	126 (59)	
>37	291 (50)	204 (55)	87 (41)	
Lactate Dehydrogenase (U/L), n (%)				0.017
≤158	307 (52)	210 (56)	97 (46)	
>158	280 (48)	164 (44)	116 (54)	
Albumin (g/L), n (%)				0.005
≤39	377 (64)	224 (60)	153 (72)	
>39	210 (36)	150 (40)	60 (28)	
Direct Bilirubin (μmol/L), n (%)				0.025
≤3.60	302 (51)	206 (55)	96 (45)	
>3.60	285 (49)	168 (45)	117 (55)	
Systemic Inflammation Response Index, n (%)				0.028
≤1.23	293 (50)	200 (53)	93 (44)	
>1.23	294 (50)	174 (47)	120 (56)	
Urea (mmol/L), n (%)				<0.001
≤5.40	303 (52)	213 (57)	90 (42)	
>5.40	284 (48)	161 (43)	123 (58)	
Total Cholesterol, n (%)				<0.001
≤4.50	295 (50)	166 (44)	129 (61)	
>4.50	292 (50)	208 (56)	84 (39)	
High-Density Lipoprotein (mmol/L), n (%)				0.002
≤1.13	306 (52)	176 (47)	130 (61)	
>1.13	281 (48)	198 (53)	83 (39)	
Low-Density Lipoprotein (mmol/L), n (%)				<0.001
≤2.89	295 (50)	166 (44)	129 (61)	
>2.89	292 (50)	208 (56)	84 (39)	
Lipoprotein(a) (mg/L), n (%)				0.031
≤185	295 (50)	201 (54)	94 (44)	
>185	292 (50)	173 (46)	119 (56)	
Albumin to Globulin Ratio, n (%)				<0.001
≤1.50	304 (52)	170 (45)	134 (63)	
>1.50	283 (48)	204 (55)	79 (37)	
Lymphocyte-to-Monocyte Ratio, n (%)				0.028
≤3.47	294 (50)	174 (47)	120 (56)	
>3.47	293 (50)	200 (53)	93 (44)	
Monocyte-to-HDL Ratio, n (%)				0.004
≤0.39	293 (50)	204 (55)	89 (42)	
>0.39	294 (50)	170 (45)	124 (58)	

In our study, we compared two groups: the CMBs group (*n* = 213) and the non-CMBs group (*n* = 374). We found significant differences (*p* < 0.05) in 30 out of the 88 analyzed variable between these two groups, with the remaining 58 variables showed no significant differences. These significant variables include demographic factors, clinical conditions, and specific biomarkers relevant to cerebrovascular health, as detailed in Table 1. This comprehensive enumeration of biomarkers, both differing and non-differing, offers a holistic understanding of the factors linked to CMBs.

### Selection of key variables

**Figure 2:**
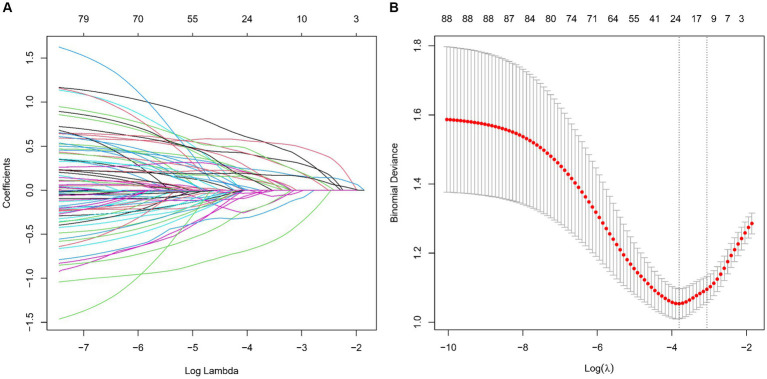
Screening of variables based on LASSO regression model in a training set of 412 patients. **(A)** Illustration of coefficient profiles for 88 potential CMBs predictors using the LASSO model. The plot shows the change in each feature’s coefficient against the log (lambda), highlighting LASSO’s shrinkage effect. **(B)** Graphical representation of LASSO regression’s cross-validation performance. The model’s effectiveness is assessed at different lambda values. λmin marks the model with optimal performance, while λ1SE indicates a more compact model.

We analyzed an array of 88 diverse variables, including demographic data, clinical history, and a range of laboratory test results. Using LASSO regression, nine significant variables emerged as pivotal predictors in our model: Albumin to Globulin Ratio, Creatinine, Gender, Homocysteine, Hypertension, Low-Density Lipoprotein, Neutrophil to HDL Ratio, Stroke, and Total Cholesterol (Table 2). This selection process, illustrated in Figures 2A,B, effectively identified variables with strong associations with CMBs, underscoring their relevance within the context of our study.

**Table 2:** Coefficients and lambda.1SE value of the LASSO regression.

Variable	Coefficients	Lambda.1SE
Albumin to Globulin Ratio	−0.0755735	0.04678
Creatinine	0.07313743	
Gender	0.07403888	
Homocysteine	0.04624465	
Hypertension	0.03754653	
Low-Density Lipoprotein	−0.0046682	
Neutrophil to HDL Ratio	0.05028032	
Stroke	0.11447028	
Total Cholesterol	−0.0185793	

This table presents the LASSO regression analysis outcomes, displaying coefficients for key variables like Albumin to Globulin Ratio, Creatinine, Gender, Homocysteine, Hypertension, Low-Density Lipoprotein, Neutrophil to HDL Ratio, Stroke, Total Cholesterol. It also includes the lambda.1SE value, crucial in model selection, indicating the model’s robustness and predictive reliability. This analysis forms a vital part of our predictive model’s development, underscoring these variables’ significance in determining the risk of CMBs.

### Multivariable logistic regression analysis

In our subsequent analysis using binary logistic regression, we incorporated the nine variables that emerged as significant in the LASSO regression. This rigorous analysis pinpointed six variables: Albumin to Globulin Ratio, Gender, Homocysteine, Hypertension, Neutrophil to HDL Ratio, and Stroke, which demonstrated a statistically significant correlation (*p* < 0.05) with CMBs, as comprehensively illustrated in Table 3. These findings suggest that these six factors independently contribute to the likelihood of CMBs occurrence. However, the inclusion of Creatinine, Low-Density Lipoprotein, and Total Cholesterol in the model did not yield statistically significant associations in this particular analysis, indicating their limited predictive value for CMBs in our study cohort.

**Table 3:** Binary logistic regression analysis.

	B	SE	OR	CI	*Z*	*p*
Albumin to Globulin Ratio	−1.097	0.269	0.33	0.2–0.57	−4.077	<0.001
Creatinine	0.521	0.321	1.68	0.9–3.16	1.623	0.105
Gender	0.915	0.278	2.5	1.45–4.3	3.293	0.001
Homocysteine	0.578	0.299	1.85	1.03–3.32	2.068	0.039
Hypertension	0.513	0.102	1.67	1.37–2.04	5.036	<0.001
Low-Density Lipoprotein	−0.291	0.463	0.75	0.3–1.85	−0.629	0.529
Neutrophil to HDL Ratio	0.55	0.268	1.73	1.02–2.93	2.053	0.04
Stroke	0.719	0.274	2.05	1.2–3.51	2.625	0.009
Total Cholesterol	−0.318	0.462	0.73	0.29–1.8	−0.688	0.491

Outlines the results of a binary logistic regression analysis aimed at identifying key predictors for Cerebral Microbleeds (CMBs), using nine variables identified by LASSO regression. Six of these variables demonstrated statistical significance and were integral to the final diagnostic model: Albumin to Globulin Ratio, Gender, Homocysteine, Hypertension, Neutrophil to HDL Ratio, and Stroke. In contrast, Creatinine, Low-Density Lipoprotein, and Total Cholesterol were not statistically significant and were excluded from the model. The odds ratios (ORs) indicate the increased or decreased risk of CMBs associated with each variable, providing crucial insights for clinical assessment and intervention.

### Development of a predictive model for CMBs

**Figure 3:**
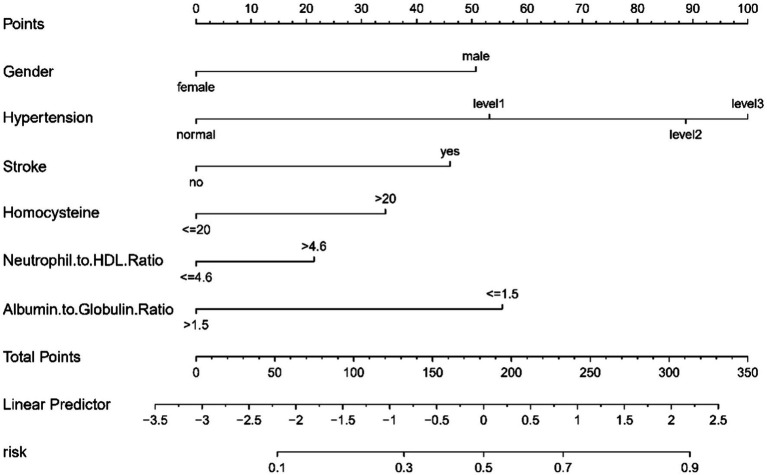
A nomogram designed to predict CMBs risk, utilizing six key indicators. Points are assigned for each factor and totaled. This total corresponds to a CMBs risk percentage on the nomogram.

Our study utilized a multivariable logistic regression framework to pinpoint crucial variables that influence the risk of CMBs. These variables, specifically Albumin to Globulin Ratio, Gender, Homocysteine, Hypertension, Neutrophil to HDL Ratio, and Stroke, were integral in crafting a comprehensive predictive nomogram (Figure 3). The nomogram functions by allocating weighted scores to each variable, with the total score for each patient corresponding to a likelihood of developing CMBs. This tool integrates biochemical indices and clinical characteristics to provide a comprehensive evaluation of CMBs risk, enhancing decision-making for healthcare providers.

### Nomogram validation

**Figure 4:**
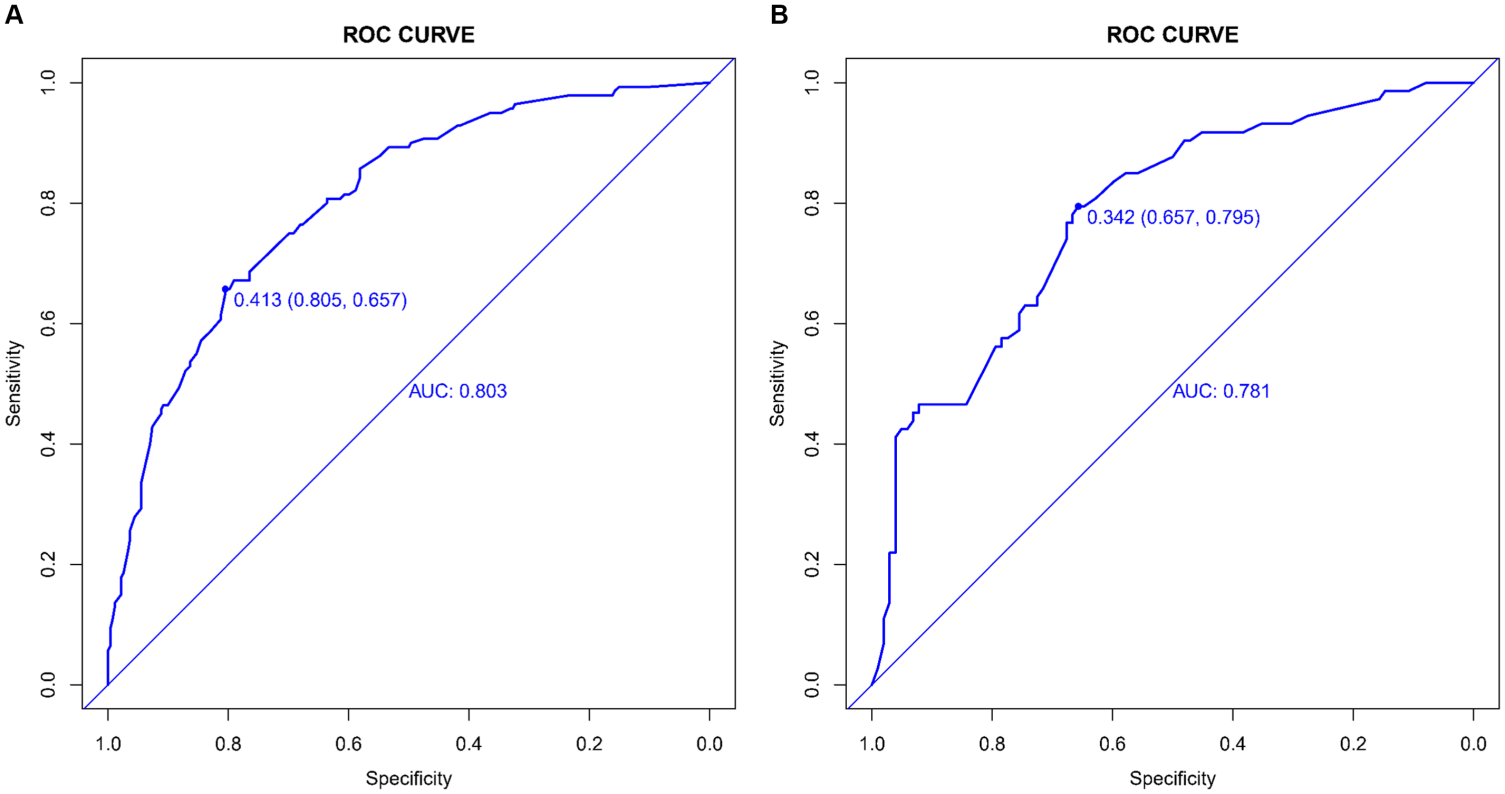
**(A,B)** ROC curves demonstrating the sensitivity and specificity of the CMBs prediction model in the training **(A)** and validation **(B)** sets.

**Figure 5:**
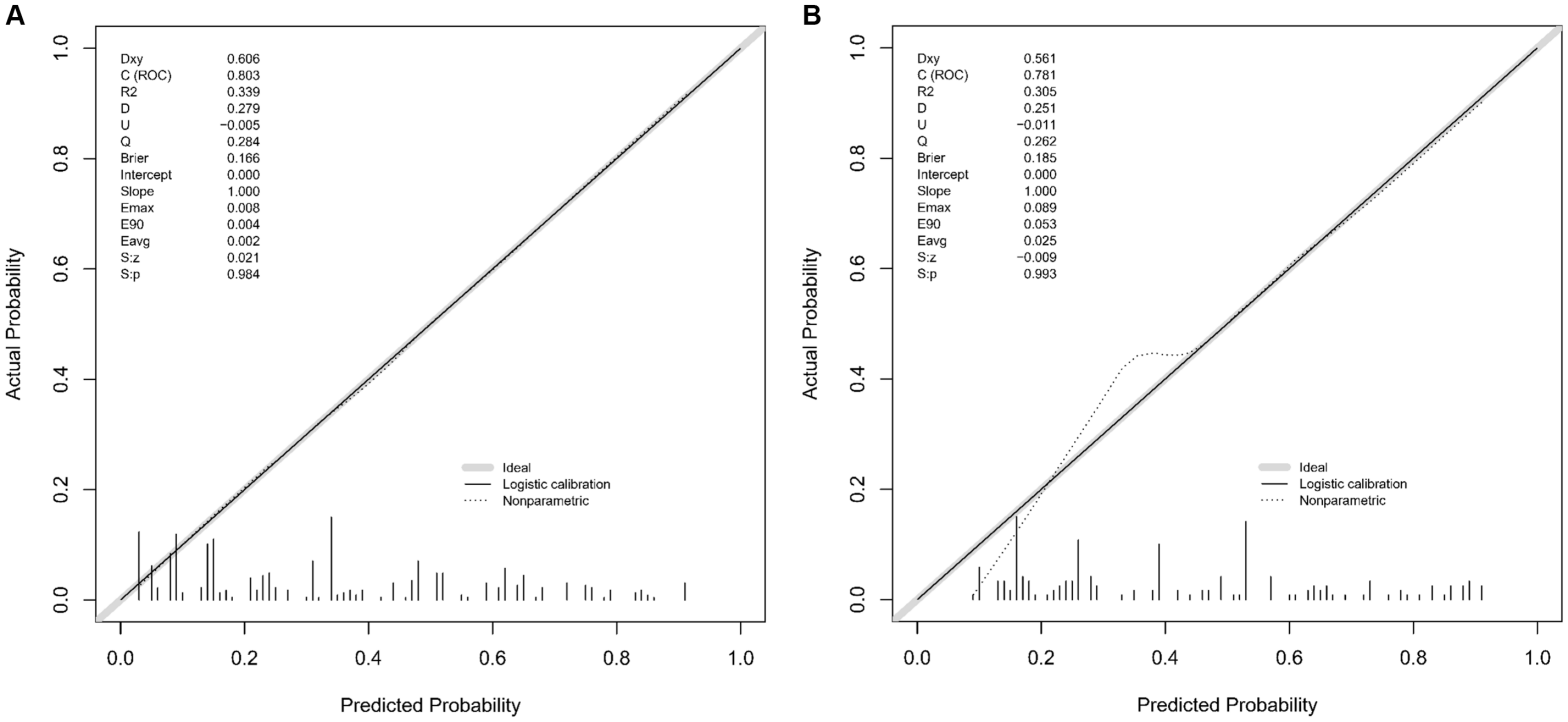
**(A,B)** Calibration plots for the CMBs predictive model in the training **(A)** and validation **(B)** sets. These plots compare the model’s predicted probabilities against actual CMBs occurrence rates.

**Figure 6:**
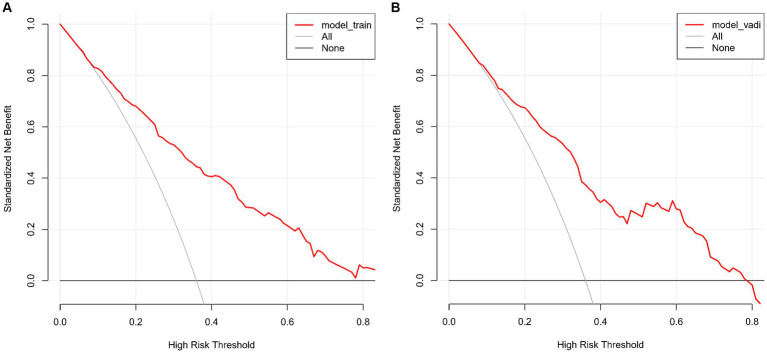
**(A,B)** Decision Curve Analysis of the CMBs prediction model for the training **(A)** and validation **(B)** sets. These graphs show the net benefit of the model’s predictions across different probability thresholds compared to two baseline strategies.

The CMB risk prediction nomogram was validated using Receiver Operating Characteristic (ROC) curves, calibration plots, and Decision Curve Analysis (DCA) in both the training and validation cohorts. In ROC analysis, the training set demonstrated an AUC of 0.803 (95% CI: 0.759–0.847), and the validation set an AUC of 0.781 (95% CI: 0.732–0.865), indicating strong discriminative ability. Sensitivity and specificity were 80.5 and 65.7% in the training set, and 65.7 and 79.5% in the validation set, respectively (Figures 4A,B). Calibration analysis revealed close alignment between predicted and observed outcomes, with Brier scores of 0.166 for the training set and 0.185 for the validation set, supporting the model’s predictive accuracy (Figures 5A,B). Decision Curve Analysis (Figures 6A,B) demonstrated the nomogram’s clinical benefit across a range of threshold probabilities. Collectively, these results affirm the nomogram’s accuracy and reliability in predicting CMBs, confirming its potential utility in clinical settings.

## Discussion

The predictive model for CMBs developed in this study represents an innovative advancement in the current research domain. We have comprehensively integrated a range of key factors: the Albumin/Globulin ratio, gender, hypertension, homocysteine levels, Neutrophil to High-Density Lipoprotein Ratio, and history of stroke. These elements collectively construct an all-encompassing risk assessment model that not only focuses on biomarkers and clinical characteristics but also considers the individual history of the patients. Notably, NHR, as a critical indicator reflecting inflammation and lipid metabolism, emerges as particularly significant in the risk assessment of CMBs.

In our predictive model for CMBs, the Neutrophil to HDL Ratio emerges as a significant predictive factor. NHR is known to reflect systemic inflammatory responses, which are closely linked to vascular endothelial dysfunction and subsequent small vessel fragility (Chen et al., 2020; Liu et al., 2022). This association is particularly relevant in cerebrovascular conditions, where chronic inflammation may accelerate vascular damage, increasing the likelihood of CMB formation. This model’s inclusion of the A/G ratio, traditionally a marker of nutritional and systemic health, aligns with recent findings in cerebrovascular research. Studies have indicated its association with functional outcomes in ischemic stroke, suggesting a broader applicability in cerebrovascular conditions, including CMBs (Maeda et al., 2019; Li et al., 2022; Yang et al., 2022). The A/G ratio’s role in ischemic stroke recovery, as reported in recent research (Yang et al., 2022; Wang et al., 2023), further substantiates its relevance in the context of CMBs. Therefore, the A/G ratio in our model reflects not only systemic health but also its potential in cerebrovascular risk stratification. The inclusion of male gender as a risk factor acknowledges gender-specific variations in cerebrovascular disease prevalence and outcomes. Studies have highlighted differences in CMBs occurrence between males and females, suggesting hormonal and biological factors might influence these disparities (Lu et al., 2021; Jensen et al., 2023). Hypertension is a critical factor in the development of cerebrovascular abnormalities, including CMBs. Our model’s emphasis on hypertension aligns with findings that consistently link elevated blood pressure with increased CMBs risk (Lu et al., 2021; Jensen et al., 2023; van Dooren et al., 2014), underscoring the need for stringent blood pressure control in high-risk individuals. Elevated homocysteine, a marker of vascular health, has been associated with an increased risk of cerebrovascular diseases. By including homocysteine levels, our model addresses the biochemical aspect of CMBs risk, reflecting findings that link homocysteine with cerebrovascular damage (Nam et al., 2019; Sudduth et al., 2013). The inclusion of past stroke history as a predictor is crucial. Previous cerebrovascular events are a strong indicator of an individual’s susceptibility to CMBs. Research has consistently shown that a history of stroke significantly elevates the risk of subsequent cerebrovascular incidents, including CMBs (Jensen et al., 2023; Shoamanesh et al., 2017). This underscores the importance of considering past cerebrovascular events in predictive modeling for CMBs. Compared to models such as Liu et al. (2024), which primarily focus on renal function indicators like blood urea nitrogen and cystatin C, our model integrates a broader range of biochemical and clinical markers to capture multiple dimensions of cerebrovascular health. Additionally, unlike automated detection models like CMB-HUNT by Suwalska et al. (2022), which rely solely on imaging data for CMB detection, our model combines imaging with clinical history and biochemical markers, addressing a critical gap in current CMB research by offering a multifaceted tool for risk prediction. In summary, our CMBs predictive model combines these factors to offer a robust tool for identifying individuals at high risk. It highlights the importance of a holistic approach in cerebrovascular risk assessment and the need for targeted interventions based on comprehensive risk profiling.

The model shows notable predictive power, which appears to be enhanced by incorporating both conventional risk factors like hypertension and novel biomarkers like NHR. This dual approach offers a more holistic understanding of the risk landscape for CMBs. In clinical practice, this model can be instrumental in identifying high-risk patients for early intervention, potentially mitigating the severe outcomes associated with CMBs. Despite its strengths, our study has limitations, primarily stemming from its retrospective design, which inherently limits causal inference and introduces the potential for selection bias. This limitation affects the model’s generalizability to broader populations, and prospective validation in diverse clinical settings and independent cohorts will be crucial to further confirm its robustness and applicability. Future work will focus on external validation studies with independent cohorts from diverse clinical and demographic backgrounds. Such studies will help ensure that the model’s predictive power and reliability extend beyond the current study population, providing a stronger foundation for its broader clinical application. Additionally, future research should integrate more biomarkers and explore genetic factors in CMBs risk, further enhancing the model’s predictive accuracy and clinical utility. While traditional risk factors like hypertension are directly actionable, novel biomarkers such as NHR and the A/G ratio, though promising, may require further investigation to establish effective intervention strategies. Unlike previous studies that have predominantly focused on imaging findings, our model offers a more comprehensive risk assessment by combining imaging with clinical history and biochemical markers. This multidimensional approach not only addresses a critical gap in current CMB research but also aligns with recent research highlighting the role of inflammatory markers and vascular health indicators in cerebrovascular disease prediction (Lu et al., 2021; Quick et al., 2021; Wan et al., 2023). By incorporating these markers alongside traditional risk factors, our model provides a more dynamic tool for risk prediction, offering a nuanced understanding of the complex interactions between systemic health, inflammation, and cerebrovascular risk.

Our model could be expanded by considering additional variables, such as genetic predispositions, lifestyle factors like diet and exercise, and environmental influences. For example, genetic polymorphisms, such as the APOE ε4 allele associated with an increased burden of cerebral microbleeds, as well as those related to lipid metabolism or inflammation could modulate the impact of NHR or the Albumin to Globulin ratio on CMBs risk. Similarly, lifestyle factors might have a direct or indirect effect on these biomarkers and clinical features, influencing the overall risk profile. Integrating these additional elements into the model could provide a more comprehensive risk assessment tool. This approach aligns with the current trend in personalized medicine, where the goal is to consider the whole spectrum of individual differences in disease prediction and management. Future research should aim at developing multifactorial models that incorporate these broader aspects, enhancing the predictive accuracy and clinical utility of CMBs risk assessment tools.

Our study successfully develops a novel predictive model for CMBs by integrating diverse clinical and laboratory parameters, including the Neutrophil to HDL Ratio, Albumin/Globulin ratio, gender, hypertension, homocysteine levels, and history of stroke. This model stands out for its holistic approach to assessing CMBs risk, highlighting the interplay between systemic health, vascular risk factors, and individual patient histories. The inclusion of both traditional and novel biomarkers, especially the Neutrophil to HDL Ratio, offers a more nuanced understanding of CMBs pathophysiology. This model represents a significant stride in personalized medicine, providing a potent tool for early identification and intervention in individuals at high risk for CMBs. While our findings are promising, they underscore the need for further research, particularly prospective studies, to validate and refine this model for broader clinical application. In clinical settings, the nomogram derived from this model offers a practical tool for patient risk stratification, allowing clinicians to quickly identify high-risk individuals and initiate early intervention with minimal workflow disruption.

## Data Availability

The original contributions presented in the study are included in the article/supplementary material, further inquiries can be directed to the corresponding author.
